# Leveraging Cancer Phenotypic Plasticity for Novel Treatment Strategies

**DOI:** 10.3390/jcm13113337

**Published:** 2024-06-05

**Authors:** Sravani Ramisetty, Ayalur Raghu Subbalakshmi, Siddhika Pareek, Tamara Mirzapoiazova, Dana Do, Dhivya Prabhakar, Evan Pisick, Sagun Shrestha, Srisairam Achuthan, Supriyo Bhattacharya, Jyoti Malhotra, Atish Mohanty, Sharad S. Singhal, Ravi Salgia, Prakash Kulkarni

**Affiliations:** 1 Department of Medical Oncology and Therapeutics Research, City of Hope National Medical Center, Duarte, CA 91010, USA; sramisetty@coh.org (S.R.); sayalurraghu@coh.org (A.R.S.); spareek@coh.org (S.P.); tmirzapoiazova@coh.org (T.M.); dando@coh.org (D.D.); jymalhotra@coh.org (J.M.); amohanty@coh.org (A.M.); ssinghal@coh.org (S.S.S.); 2 City of Hope Atlanta, 600 Celebrate Life Parkway, Newnan, GA 30265, USA; dhivya.prabhakar@coh.org; 3 City of Hope Chicago, 2520 Elisha Avenue, Zion, IL 60099, USA; episick@coh.org; 4 City of Hope Phoenix, 14200 West Celebrate Life Way, Goodyear, AZ 85338, USA; sagun.shrestha@coh.org; 5 Center for Informatics, City of Hope National Medical Center, Duarte, CA 91010, USA; sairam.achuthan@gmail.com; 6 Integrative Genomics Core, City of Hope National Medical Center, Duarte, CA 91010, USA; sbhattach@coh.org; 7 Department of Systems Biology, City of Hope National Medical Center, Duarte, CA 91010, USA

**Keywords:** adaptive therapy non-genetic mechanism, persisters, phenotypic plasticity

## Abstract

Cancer cells, like all other organisms, are adept at switching their phenotype to adjust to the changes in their environment. Thus, phenotypic plasticity is a quantitative trait that confers a fitness advantage to the cancer cell by altering its phenotype to suit environmental circumstances. Until recently, new traits, especially in cancer, were thought to arise due to genetic factors; however, it is now amply evident that such traits could also emerge non-genetically due to phenotypic plasticity. Furthermore, phenotypic plasticity of cancer cells contributes to phenotypic heterogeneity in the population, which is a major impediment in treating the disease. Finally, plasticity also impacts the group behavior of cancer cells, since competition and cooperation among multiple clonal groups within the population and the interactions they have with the tumor microenvironment also contribute to the evolution of drug resistance. Thus, understanding the mechanisms that cancer cells exploit to tailor their phenotypes at a systems level can aid the development of novel cancer therapeutics and treatment strategies. Here, we present our perspective on a team medicine-based approach to gain a deeper understanding of the phenomenon to develop new therapeutic strategies.

## 1. Introduction

Cancer is a global health-care burden and a leading cause of death worldwide [1]. In fact, in the next 25 years (by 2050), the estimated global economic cost of all cancers is projected to skyrocket to >USD 25 trillion [2]. The top five cancers projected to have the highest economic costs globally are tracheal, bronchus, and lung cancer (15.4%), colon and rectal cancer (10.9%), breast cancer (7.7%), hepatocellular carcinoma (6.5%), and leukemia (6.3%) [2]. In the US alone, >2 million new cancer cases and >600,000 cancer deaths are projected to occur in 2024 [3]. These staggering statistics are a stark reminder of the burgeoning health-care issue associated with cancer worldwide. They also underscore the dire need to come up with effective therapeutics and treatment strategies to treat this dreadful malady.

Every year, dozens of new cancer drugs or drug combinations are approved for clinical use that displace drugs previously approved, and represent the standard of care for their indication or are approved as later-line drugs in second-, third-, or later-line settings [4]. Nonetheless, despite the initially striking response rate seen in the clinic, most, if not all, patients begin to show a muted response to the drug and eventually go on to develop resistance and disease progression [5]. Sotorasib (AMG510), a small-molecule compound that selectively targets the mutant KRAS G12C, which until just a few years ago was deemed “undruggable”, is a good case in point. Despite the initial euphoria following its approval by the FDA, the duration of response for most patients is disappointing: the median progression-free survival was found to be only 6.3 months [6] to 6.8 months [7], underscoring the nettlesome issue of drug resistance and how it has inadvertently emerged as a major impediment in treating cancer. Several studies have attempted to synergize mutant-specific KRAS small-molecule inhibitors with other therapeutic approaches to improve outcomes. For example, it is known that RAS-mutant tumor cells can create an immunosuppressive tumor microenvironment (TME). Therefore, they can potentially increase the susceptibility of these tumors to immunotherapies, providing a good rationale for combining RAS-inhibitory drugs with immune checkpoint blockade (ICB). However, even this strategy has proven challenging due to the inherent phenotypic plasticity of cancer cells [8].

Perhaps one of the major reasons for the persistence of the issue of drug resistance could be that drug resistance per se is more nuanced than is typically perceived and defined [9]. Until recently, drug resistance, whether innate or acquired, was thought to arise primarily through random genetic mutations and the subsequent expansion of mutant clones via Darwinian selection. Hence, the phenomenon has traditionally been approached from a purely reductionist, gene-centric perspective. Furthermore, under current treatment protocols, the patient is treated with the drug or drug combinations and doses through multiple cycles using the maximum tolerated dose (MTD). The underlying assumption of this therapeutic strategy, the so-called continuous therapy, is that a tumor can be rapidly eradicated, and thus it can preclude the evolution of resistant clones and their dissemination to distant locations. However, this rationale is an oversimplification and in fact is counterintuitive. Ironically, continuous treatment at the MTD favors the resistant population because it not only strongly selects for adapted phenotypes but also eliminates all potentially competing populations [10].

**Figure 1:**
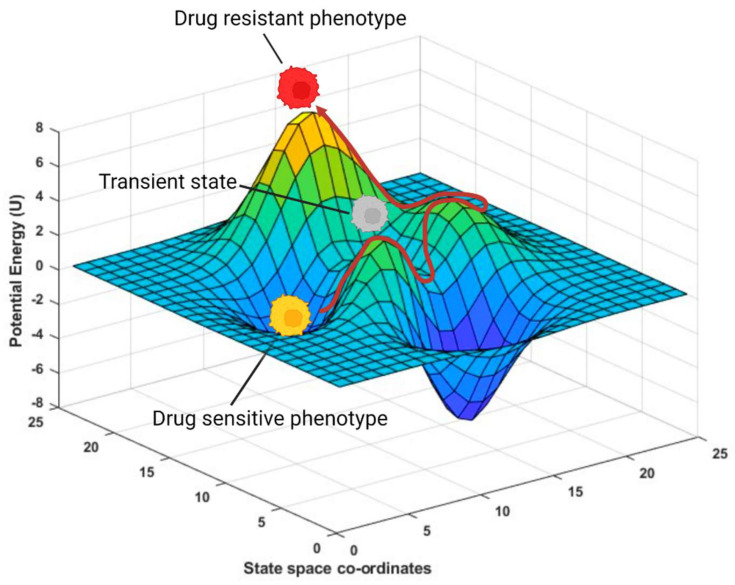
Cancer network state space as an energy landscape. The energy landscape shows cancer cell states over time with drug exposure. The x–y plane represents state space coordinates, and the z-axis represents potential energy *U*, indicating cell fate stability. Initially, stable drug-naïve cancer cells occupy fixed points and low-energy attractors. Upon drug treatment, cancer cells transition from the stable phenotype (yellow cell) towards other high-energy yet less stable attractors to increase their fitness (red cell). During this transition, cancer cells may traverse transient states (gray cells).

Accumulating evidence indicates that drug resistance does not occur through mutations acting alone. Non-genetic mechanisms, including epigenetic modifications and protein interaction network (PIN) rewiring leading to phenotypic switching, can also impact how a cancer cell acquires the ability to evade the harmful effects of drug treatment. In this scenario, PIN rewiring leads to an intermediary drug-tolerant state that is reversible. In response to chronic drug treatment, however, the PIN is frustrated and mutations in specific proteins that occupy key positions in the PIN are introduced to relieve frustration. This results in irreversible drug resistance [10,11]. In fact, in bacteria too, it has been observed that in the short-term, the microbes acclimate to stress through phenotypic plasticity, but in the longer term, they adapt to stress genetically [12]. Additionally, computational models investigating stochastic phenotypic switching in bacteria [13] showed that when a genetic mutation renders the population less fit, switching to an alternative phenotype with higher fitness gives the population sufficient time to develop compensatory mutations, demonstrating that phenotypic switching can reduce the time to adaptation by orders of magnitude. Together, these observations lend further credence to the idea that phenotypic plasticity is of fundamental importance in developing drug resistance and speeding up the evolution of bacteria and cancer cells [14] (Figure 1). Furthermore, group behavior emerging from phenotypic plasticity can sustain a heterogeneous cancer cell population with multiple interchangeable phenotypes, producing temporary drug tolerance and facilitating the initiation and progression to permanent drug resistance [4,7,10,11,15].

Here, we discuss the virtues of a team medicine approach to gain a deeper understanding of how phenotypic plasticity enables cancer cells to evade harmful drug effects. We highlight how this interdisciplinary and integrated systems-level approach has not only uncovered the phenotypic plasticity of cancer cells as the major impediment in treating the disease but has also revealed how it may be leveraged to develop new treatment strategies.

## 2. Phenotypic Plasticity Is an Emergent Property of Cancer Cells

From a physics perspective, a cancer cell, like all living forms, is a complex adaptive system (CAS). CASs comprise an ensemble of dynamic networks of interactions (or agents); however, the behavior of the ensemble is difficult to predict based on the behavior of the individual components. Furthermore, CASs are self-organizing systems with emergent behavior capable of adapting to the changing environment and increasing their survivability as a macrostructure. In other words, they strive to increase their fitness as they traverse the rugged fitness landscape by “making” informed decisions (Figure 1). Indeed, it has been suggested that analogously to a computer, a living cell such as a cancer cell comprises hardware and software collectively referred to as the “wetware”, and is attuned to logic gates capable of “making” decisions [16]. Although the idea that a cell (or a protist) can make decisions may sound ludicrous, perhaps even eerie, emerging evidence indicates that indeed cells can “anticipate” and even “learn” from the experiences that they seem to recall when needed later [17]. Thus, a living cell may be perceived as being analogous to a perceptron (or McCulloch–Pitts neuron), a single-layer neural network that integrates input assigned with a weighted matrix and computes an output such as a decision to switch phenotypes. These tantalizing revelations have not only put phenotypic plasticity in perspective but have also put scientists at the vanguard of a new field called basal cognition, which embodies fundamental processes and mechanisms that enabled protists to monitor or track environmental states and act appropriately to ensure survival and reproduction long before the metazoan nervous systems developed during evolution [18,19,20,21,22,23,24].

Thus, it follows that the remarkable ability of cancer cells to adapt and survive in the face of therapeutic challenges is not simply a byproduct of random mutations, but rather an emergent property of a CAS. Therefore, cancer cells can be considered as decision-makers actively surveying and responding to their microenvironment. This perspective reshapes our understanding of phenotypic plasticity in cancer, not as a series of isolated events but as a cohesive and evolved strategic response, integral to the cellular network’s adaptive landscape. Cancer cells leverage phenotypic plasticity and engage in a dialogue with their surroundings to ensure survival and propagate resistance, thus presenting novel challenges as well as opportunities for therapeutic intervention.

Cancer cells adapt to changing conditions by virtue of their plasticity. Epithelial–mesenchymal plasticity (EMP) is one of the well-studied axes of plasticity [25]. This bidirectional process is a landscape that includes multiple hybrid E–M phenotypes, which possess the properties of both epithelial and mesenchymal cells, thus conferring them with properties like enhanced migration and therapy evasion, which are associated with poor patient prognosis [26]. Factors like NFATc [27], SLUG [28], KLF4 [29], and ELF3 [30] have been identified to play crucial roles in regulating EMP.

## 3. Cancer Cells Explore Non-Genetic Mechanisms to Increase Their Fitness

One of the first reports uncovering a non-genetic mechanism by which lung cancer cells can evade the toxic effects of drugs showed that individual cells in a population transiently assume a reversibly drug-tolerant state [31]. This subpopulation, which showed >100-fold reduced drug sensitivity, maintained viability via engagement of IGF-1 receptor signaling and epigenetic chromatin modifications sculpted by the histone demethylase KDM5A/JARID1B. More importantly, this drug-tolerant phenotype was found to be transiently acquired by a tiny fraction of individual cells within the population, underscoring the dynamic regulation of phenotypic heterogeneity in drug tolerance [31].

Soon after, another study on melanoma reported the identification of a small subpopulation of slow-cycling cancer cells using KDM5B/Jarid1B as a biomarker [32]. Knocking down JARID1B leads to an initial acceleration in tumor cell growth followed by exhaustion, suggesting that the JARID1B-positive subpopulation is essential for sustained tumorigenesis. Strikingly, JARID1B expression was highly dynamic, with JARID1B-positive cells becoming JARID1B-negative and vice versa [32]. While these tantalizing observations provided credence to the idea that non-genetic mechanisms may also play an important role in the emergence of drug resistance, they also underscored the fact that a drug-tolerant state is transient and can be stochastically assumed by any individual cell in the population.

This remarkable finding also has other far-reaching implications, for example, on discovering reliable biomarkers and genome-wide association studies (GWAS) that compare the genomes from different cancer patients to find genetic markers associated with a particular phenotype or risk of the disease, which some believe may be the holy grail. Although it would seem quite logical to identify biomarkers and/or genetic markers that can be used to identify the disease at an early stage, for disease prognostication, or to discern patients who are likely to respond to a specific drug from those who are unlikely, finding such reliable biomarkers and genetic markers has been challenging [33,34,35,36,37,38,39]. Perhaps the obvious reason that has thus far been ignored is the inevitable phenotypic heterogeneity that arises stochastically and non-genetically [5,40].

The lure of the cancer/testis antigens (CTAs) as novel biomarkers is yet another example of how phenotypic plasticity is leveraged by cancer cells [41,42,43]. The CTAs, particularly those located on the X chromosomes and referred to as CT-X antigens, are a heterogeneous group of proteins whose expression is typically confined to the germ cells. However, they are aberrantly expressed in many cancers. Since they are sequestered from the immune system in the normal adult, the CTAs, especially the CT-X antigens, some of which are remarkably germline-specific, are ideal candidates for biomarkers as well as for immunotherapy [44,45,46]. Nonetheless, thus far, they have met little success in the clinic, likely due to their dynamic expression patterns, which result in phenotypic heterogeneity in the population. Indeed, JARID1B is also a CTA [47], underscoring the hurdles posed by the cancer cell’s plasticity. Even if they are approved for clinical use, the emergence of resistance, as seen in the evitable case of small-molecule drugs, would be a major concern. A recent study showing how resistance to antibody-dependent cellular cytotoxicity (ADCC) is impacted by phenotypic plasticity [48] lends further credence to this valid concern. Interestingly, that study also showed that immune-resistant clones regained sensitivity upon the withdrawal of therapeutic (ADCC) pressure, implying phenotypic plasticity and reversibility [48].

These and several other subsequent studies [9,49,50,51] are in striking contrast to the “central dogma of cancer”. That is, cancer is a genetic disease, and pre-existing clones of cancer cells with advantageous mutations that arise fortuitously are selected in response to drug treatment giving rise to innate drug resistance (Figure 1). This assumption, that cancer is a purely genetic disease, further underscores the deterministic nature of the disease. However, other studies on melanoma using the same drug, namely, vemurafenib, which targets BRAFV600E, concluded that resistance can emerge via genetic (mutation) [52] as well as non-genetic mechanisms [53]. These contrasting observations caused a conundrum that begged reconciliation. The conundrum was addressed by Mahmoudabadi et al. who proposed the “conformational noise” hypothesis highlighting the role of intrinsically disordered proteins (IDPs) in cellular stress response [54].

## 4. Intrinsically Disordered Proteins, Conformational Noise, and Phenotypic Switching

**Figure 2:**
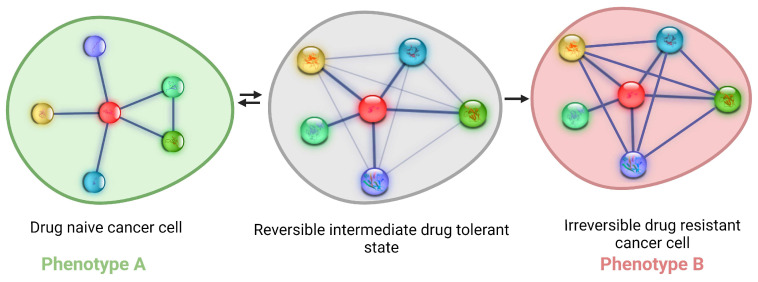
Phenotypic switching from drug-sensitive to drug-resistant phenotype via protein interaction network (PIN) rewiring. Upon exposure to a drug, sensitive cells Phenotype A, attempt to evade the harmful drug effects by rewiring their PIN. This non-genetic mechanism results in a reversible drug-tolerant state. However, prolonged drug treatment leads to network frustration, which leads mutations in certain “hot spot” loci to alleviate this frustration. This results in the emergence of an irreversible drug-resistant state, referred to as phenotype B.

IDPs are proteins or regions within structured proteins referred to as intrinsically disordered regions (IDRs) that lack rigid 3D structures. Instead, they exist as conformational ensembles, and while some IDPs/IDRs can fold upon interacting with partner proteins or other molecules in the cells or upon post-translational modification(s), several IDPs/IDRs remain unstructured even when interacting [55]. Due to the inherent structural plasticity and a high degree of malleability, IDPs/IDRs can promiscuously interact with multiple partners. These interactions form cellular PINs that adopt a scale-free architecture and act as the principal conduits of information flow in the cell (Figure 2).

Because of their plasticity, IDPs typically tend to occupy hub positions in cellular PINs. Furthermore, their conformational plasticity can be further impacted due to their propensity to undergo post-translational modifications such as phosphorylation, which is thought to contribute to “conformational” noise, which is distinct from the well-recognized transcriptional noise. Therefore, the conformational dynamics of IDPs in response to a specific input, such as stress, contributes to increased noise in the system and hence an increase in stochastic and “promiscuous” interactions. These interactions lead to the activation of latent pathways by rewiring the PIN to yield a new PIN configuration that produces the optimal output, underscoring the critical role of IDPs in regulating information flow (Figure 2). Indeed, evidence accumulated to date lends further credence to this hypothesis as well as providing a conceptual framework to explain how IDP conformational dynamics and conformational noise impact cellular decision-making [2,17,56,57].

While the conformational noise hypothesis explains how the PIN rewiring heuristic can uncover latent pathways and cause phenotypic switching (Figure 2), an important question arises: Is information transmitted in the reverse direction from phenotype to genotype, and if so, how is it inherited across generations? It is now well-recognized that epigenetic modifications can be transmitted transgenerationally [58]. Consistent with these observations, several chromatin modifiers are IDPs, suggesting that PIN rewiring could result in heritable epigenetic changes [59,60,61]. Since the overwhelming majority of transcription factors and chromatin modifiers are IDPs, it is highly plausible that the ripple effect of transcriptional noise and conformational noise could act in conjunction to make changes in the genome, facilitating transgenerational information transfer much like the phenomenon of “soft inheritance” [62].

## 5. Phenotypic Plasticity, Non-Genetic Phenotypic Heterogeneity, and Bet Hedging

The idea that phenotypic plasticity and non-genetic mechanisms play important roles in cancer, including metastasis and drug resistance, was suggested a decade ago [54], and is now well supported by several lines of experimental evidence [57,63,64,65]. In fact, it is now clear that phenotypic plasticity and non-genetic mechanisms are not only critical but are also leveraged as a bet-hedging strategy in cancer [47,66,67,68,69]. Furthermore, the realization that phenotypic plasticity and non-genetic mechanisms can also account for a “persister” population contributing to drug resistance in cancer further underscores their importance in the disease pathology [70,71,72,73,74,75,76,77].

Drug-tolerant persisters are not specific to cancer per se: they are also seen in bacteria and appear to arise stochastically and exist as dormant cells in the population [1,70]. Indeed, like bacteria, cancer cells can switch on cell autonomous traits in response to stress [67,78,79]. Furthermore, because of the IDP conformational noise, every cell in the population can stochastically sample the PIN space. Because of this heuristic, drug-tolerant persisters can appear a priori even before lethal drug treatment, uncovering their “bet-hedging” strategy, an evolutionary strategy adopted by the population that aims to maximize the fitness of an isogenic or clonal population in dynamic environments through phenotypic heterogeneity [80]. Consistent with this thinking, bistability (existence of two distinct subpopulations that may reversibly transition to one another) in PINs driving persistence has been proposed to give rise to persisters [81,82,83] (Figure 2).

Persisters are slow-growing tolerant cells that can arise stochastically (stochastic persistence or bet hedging, as mentioned above) or in response to a trigger (triggered persistence) and thereby give rise to tolerant cells that play a dominant role in the emergence of drug resistance [84]. While persistence usually refers to a subpopulation of cells, tolerance is the general ability of the population to survive longer treatments by phenotypic rewiring. More importantly, both these states are reversible, implying a causative role for non-genetic and epigenetic mechanisms. Thus, true resistance involves irreversible genetic alterations that enable the emergence of resistance-causing mutations via an intermediate state of tolerant cells in the population [85].

## 6. Leveraging Group Behavior of Cancer Cells to Develop New Therapeutic Strategies

**Figure 3:**
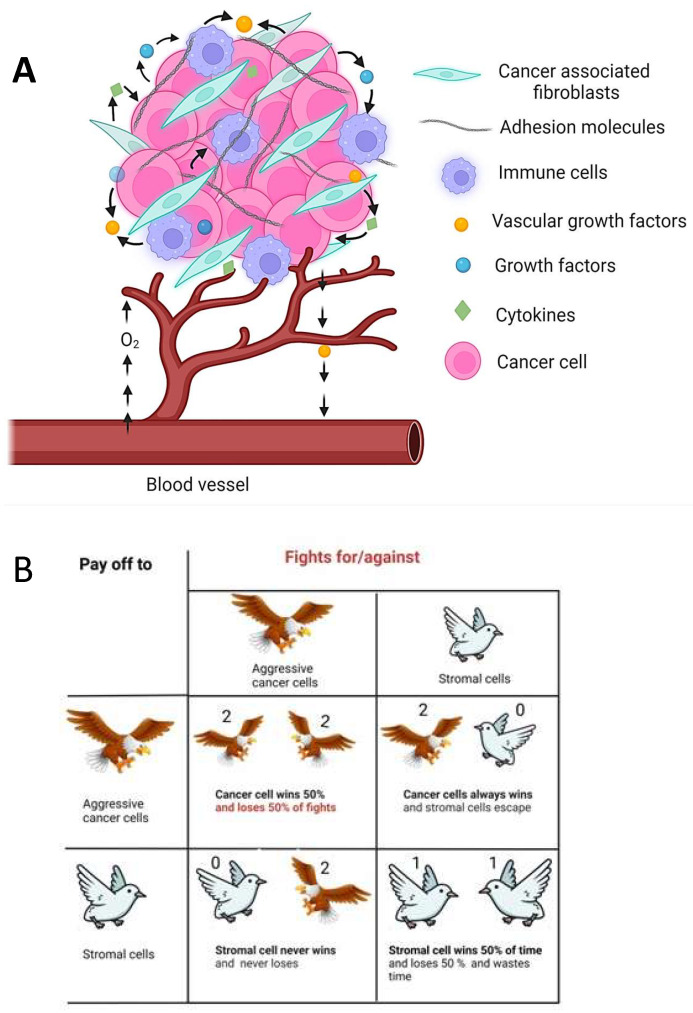
Schematic representation illustrating the cellular interactions within the tumor microenvironment (TME). (**A**) The TME is comprised of diverse cell types, including cancer cells, cancer-associated fibroblasts (CAFs), immune cells (e.g., T cells, macrophages), and endothelial cells. Cancer cells exhibit group behavior facilitated by cell–cell communication, leading to both competition and cooperation among phenotypically distinct cancer cell populations for the nutrients available. Arrows represents interactions among various cell types drive tumor progression and therapeutic response. (**B**) The pay-off matrix represents the outcomes of different strategic choices made by cancer cells. The aggressive cancer cell is represented by the hawk and the stromal cell by the dove. In this game, cancer cells can adopt two main strategies. 1. Hawk Strategy: Represents aggressive behavior or competition. When two hawks interact, they engage in a conflict that may result in a high-cost outcome. 2. Dove Strategy: Represents cooperative behavior or non-aggression. When two doves interact, they avoid conflict, resulting in a low-cost outcome. Each cell in the matrix corresponds to the pay-off associated with the interaction between two cells adopting different strategies.

Group behavior is enabled via cell–cell communication and manifests as competition and cooperation among phenotypically different cancer cells. Collective behavior is often an emergent property of self-organizing systems. Furthermore, in addition to malignant cells, tumors harbor several other “supporting” cells, such as the cancer-associated fibroblasts (CAFs), immune cells of various types, as well as endothelial cells. CAFs are an abundant and active stromal cell population in the tumor microenvironment (TME), and function as the signaling center and a remodeling machine to aid the creation of a desmoplastic tumor niche [86,87] (Figure 3A). Together, these supporting cells create an ecosystem that enables the malignant cell population to grow and flourish by producing growth factors and proinflammatory cytokines to promote angiogenesis [88] (Figure 3A). Such processes impose costs and benefits on the participating cells that may be conveniently recast in the form of a game pay-off matrix (Figure 3B). Thus, tumor progression and dynamics can be described in terms of evolutionary game theory (EGT), which provides an elegant conceptual framework to capture the frequency-dependent nature of ecosystem dynamics. It also serves to discern the games cancer cells play by either cooperating or competing in the absence or presence of stress (selective pressure) (Figure 3B) or even switching phenotypes in the presence of stress. Therefore, treatment strategies that consider the tactics cancer cells adopt to deal with therapies could potentially delay or even prevent drug tolerance and eventually drug resistance [89,90,91,92]. Thus, non-genetic mechanisms involving group behavior guide cell fate decisions in the emergence of drug resistance in cancer.

As mentioned above, existing protocols aim to maximize therapeutic potential by administering a continuous therapy strategy. However, it is now unequivocal that such a strategy can be counterintuitive and may encourage the emergence of resistant clones, albeit inadvertently. As a result, new treatment strategies, referred to as intermittent or adaptive therapy, and other approaches targeting tumor cell cooperation have emerged as promising alternatives [15,78,93,94,95]. Here, the rationale is to administer a lower dose, much lower than the MTD, so that a stable disease that remains drug-sensitive is achieved. Indeed, a lower therapeutic dose also positively impacts the quality of life of the patient and shows fewer side-effects. The rationale for the newer strategies is based on the fact that cancer cells leverage a bet-hedging strategy to evade the harmful effects of drug treatment. Nonetheless, treatment strategies that are cognizant of cancer’s ruse can turn the tables against the disease. Before treatment initiation, drug-resistant cells bear lower fitness compared to their sensitive kin [96]. However, certain subclones can gain a fitness advantage upon treatment, especially with the MTD, and therefore, by keeping the drug doses low and intermittent, the proliferation of resistant subclones can be delayed, if not completely precluded.

Despite some success achieved by adaptive therapy strategies, there may be situations where bet hedging may depend on the robustness of non-genetic mechanisms underlying bet hedging, rather than competition between sensitive and resistant clones. In such cases, perhaps combination therapies including cytotoxic drugs and inhibitors targeting phenotypic plasticity may be more efficacious in delaying the emergence of drug resistance [97,98,99].

In addition to adaptive therapy, other therapeutic strategies have been developed that are related to the “public goods game” in EGT. Such strategies specifically target tumor cell cooperation within the tumor microenvironment (TME), which comprises cancer cells and stromal cells that benefit from each other. Thus, cells that secrete beneficial factors are referred to as cooperators and those that benefit from cooperators without incurring the cost of producing any benefit are considered defectors. The latter would have greater growth within the tumor [100,101]. Mathematical models simulating the effect of selectively suppressing the benefactor cells or introducing defector cells into the tumor population have been developed to discern the dynamics of competition and cooperation among different subclones, as well as the robustness of resistance arising from non-genetic mechanisms. These efforts have resulted in alternate and efficient strategies to suppress the tumor compared to conventional strategies employing continuous therapy [93,102,103]. Such strategies can also be leveraged to shift the phenotypic composition of the tumor to a desirable equilibrium that is more manageable clinically.

## 7. Conclusions

To celebrate its 40th anniversary, *Cell* brought out a special issue in which Robert Weinberg wrote a truly insightful article: “Coming full circle—from endless complexity to simplicity and back again” [104]. An excerpt from this article states, “Those who have participated in cancer research during this period have witnessed wild fluctuations from times where endless inexplicable phenomenology reigned supreme to periods of reductionist triumphalism and, in recent years, to a move back to confronting the endless complexity of this disease”, and from a scientist with more than six decades of cancer research experience, this further underscores the ethos of the present paper.

Nonetheless, we are not out of the woods as yet and more work needs to be done. Despite the promise and excitement, it is imminent that a deeper understanding of the newer therapeutic strategies and of any side effects, especially in the case of drug combinations, is warranted. For example, in one study, it was found that in tumors sensitive to two or more drugs, the simultaneous application of these drugs resulted in cells resistant to both therapies. However, when administered one at a time, the emergence of double-resistant cell clones was delayed, implying that subpopulations of cells were sensitive to one or the other drug [105]. On the other hand, a different study found that concurrent targeting of multiple kinases, rather than a single kinase, showed complete inhibition of tumor growth [106]. More importantly, however, the latter strategy was ineffective if continuous therapy was given, but was effective when intermittent therapy was administered, alluding to the possibility that the striking inhibitory effect may have been due to the inability of the tumor cells to adapt themselves to the highly dynamic fitness threshold imposed by selection.

From the foregoing, it follows that a “team medicine” approach, involving an interdisciplinary team of basic research scientists working together with clinicians, can lead to new therapeutic strategies based on new thinking. Adopting such non-conventional strategies into clinical protocols could potentially enhance patient outcomes in terms of both progression-free and overall survival rates. Perhaps it may not be utterly preposterous to say that new drugs, often against the same target, but billed as “next generation”, either because they are more efficacious or have potentially milder side effects, may not be the answer to effective cancer treatment, as there is no guarantee the new drugs will not lead to drug resistance. It is a foregone conclusion that the cancer cells will figure out a way to become resistant to a given drug. Instead, devoting increased effort to understanding drug resistance appears to be an urgent need in order to address cancer. Perhaps acknowledging this fundamental problem may help avert a situation akin to the tragedy of the commons [107], which seems quite imminent in light of the diminishing return on investment of billions and billions of dollars every year on oncology drug development worldwide.

Over the past 4 billion years since the last universal common ancestor, its descendants, including cancer cells, have honed their ability to withstand perturbations to their environment, and as a result are adept at dealing with the challenges they face. In fact, as Max Delbruck said, “Any living cell carries with it the experiences of a billion years of experimentation by its ancestors.” [108]. While the chances of a pre-existing mutation (innate resistance) in the population are non-zero, it is an expensive proposition, and the probability, at least in theory, would seem infinitely small. Nonetheless, contrary to the prevailing wisdom that mutations occur purely stochastically (by chance) at constant, gradual rates, cancer cells possess mechanisms of mutagenesis that are upregulated by stress responses [109] (occur due to necessity). Thus, a cancer cell would first develop drug tolerance via non-genetic mechanisms and eventually resistance via specific mutations induced by chronic stress such as drug treatment (acquired resistance). On the other hand, the presence of a small fraction of cells in the population that act as persisters and give rise to cells that can evade a drug’s effects is a proven bet-hedging strategy that is seen in cancer. Importantly, persisters are not genetically predetermined, but arise stochastically via non-genetic mechanisms.

**Figure 4:**
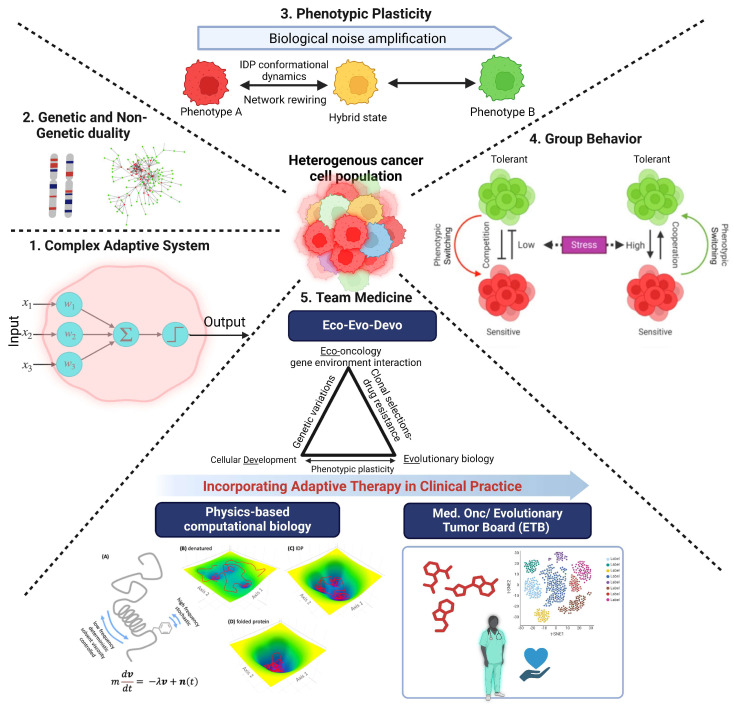
A schematic illustrating the ethos of the systems biology-based team medicine approach to address drug resistance in cancer. Clockwise as follows. (**1**) Cellular decision-making is analogous to a perceptron that computes a calculated output based on weighted inputs. (**2**) Genetic/non-genetic duality: both genetic (mutations) and non-genetic mechanisms (IDP conformational noise, gene expression noise, epigenetic modifications) contribute to the evolution of drug resistance in cancer. (**3**) Phenotypic plasticity, facilitated by protein interaction network (PIN) rewiring, stands out as a crucial mechanism of drug resistance. This process allows cancer cells to dynamically adjust their phenotypic characteristics in response to drug pressure, leading to the emergence of drug-resistant phenotypes via an intermediate drug-tolerant state. (**4**) Cancer cells exhibit complex group behavior, wherein they compete and cooperate under stress. This dynamic interplay among cancer cells in the population can lead to a collective shift from sensitive to resistant phenotypes, contributing to the development of drug resistance. Adapted from Ref. [113]. (**5**) Addressing the challenges of drug resistance in cancer could benefit from the collaborative efforts of multidisciplinary teams comprising physicists, computational biologists, evolutionary biologists, chemists, biologists, and clinicians that draw on complementary strengths and expertise, yet are brought together by very similar ideas. (**Panels A–D**) in (**5**) (Team Medicine) is adapted with permission from Ref. [17].

Although cancer has traditionally been considered a disease related to aging, recent data indicate a rise in the incidence of the disease in young adults in their 40s (early onset) [110,111]. Indeed, according to some models based on global data, the number of early-onset cancer cases will rise ~30% between 2019 and 2030 [112]. Hence, it is imperative that we discover treatment strategies for these patients to extend overall survival with a reasonable quality of life for decades, not just a few months. Given the amazing evolvability of cancer cells that resulted from the countless environmental challenges they encountered, adaptive and dynamic therapeutic strategies are more likely to prove effective than continuous therapy at the MTD in treating this pernicious disease (Figure 4).
